# Effects of Regular Recreational Exercise Training on Serum ANGPTL3-Like Protein and Lipid Profile in Young Healthy Adults

**DOI:** 10.1515/hukin-2015-0113

**Published:** 2015-12-30

**Authors:** Ewa Smol, Barbara Kłapcińska, Katarzyna Kempa, Artur Fredyk, Andrzej Małecki

**Affiliations:** 1 Department of Physiological and Medical Sciences, The Jerzy Kukuczka Academy of Physical Education in Katowice; 2 Department of Individual Sports, The Jerzy Kukuczka Academy of Physical Education in Katowice; 3 Department of Physiotherapy in Neurological and Musculoskeletal Disorders, The Jerzy Kukuczka Academy of Physical Education in Katowice

**Keywords:** angiopoietin-like protein 3, serum HDL-cholesterol, recreational physical activity

## Abstract

Evidence of the role of ANGPTL3, a liver-secreted glycoprotein, in serum lipid turnover, led us to hypothesize that this protein may be involved in modification of the lipid profile induced by exercise-training. Given the lack of data regarding this issue, the main goal of the present study was to investigate the effects of regular participation in a recreational physical activity program on serum ANGPTL3 and selected lipid profile measures in young, apparently healthy female and male adults. We compared serum ANGPTL3, lipid profile measures, common lipid ratios, the Atherogenic Index of Plasma (AIP) and glucose in fasting blood samples derived from 22 active physical education students including active females (AF, N=6) and males (AM, N=16) with samples from 28 relatively sedentary age-matched peers, including female (SF, N=9) and male (SM, N=19) individuals not involved in any regular physical conditioning program. Despite high inter-individual variability of serum ANGPTL3, there was a general tendency toward higher serum ANGPTL3 and HDL-C in women compared to men, but without significant differences related to their physical activity status. Based on both routine lipid profile measures and lipid ratios, all participants had normal lipid profiles, normal glycemia, as well as favorable anthropometric indices not suggesting increased cardiometabolic risk. However, lower levels of the TG/HDL-C ratio and AIP in physically active compared to relatively sedentary participants, reflecting the predominance of large, buoyant LDL particles, strongly support the view of beneficial health-promoting effects of regular participation in recreational sport activities.

## Introduction

An abnormal blood lipid profile, characterized by elevated levels of total cholesterol, low density lipoprotein-cholesterol (LDL-C) and triacylglycerols (TG), and a low level of high-density lipoprotein-cholesterol (HDL-C), is recognized as one of the major risk factors for coronary heart disease (CHD) (Ingelsson et al., 2007) and a stroke (Go et al., 2014). Other contributing risk factors include (inter alia) high blood pressure, impaired glucose tolerance, obesity and physical inactivity (Go et al., 2014). It is well established that regular physical activity induces metabolic adaptations characterized by improvement of the blood lipid profile and reduced CHD risk (Durstine et al., 2001). The magnitude of these beneficial changes in the lipid profile, typically recorded in endurance athletes, is related to both intensity and duration of exercise (Durstine et al., 2001). In available literature, there are reports indicating that regular recreational physical activity may also lead to improvement of the blood lipid profile (Kłapcińska et al., 2008; Popovic et al., 2010; Scheers et al., 2007).

Among many factors mediating the exercise-induced changes in plasma HDL-C and TG concentrations, an important role is attributed to some lipolytic enzymes, such as lipoprotein lipase (LPL) and hepatic lipase (HL) (Leaf, 2003). There is evidence that activity of LPL, a key enzyme hydrolyzing the TG portion of TG-rich lipoproteins, increases in the exercised skeletal muscles, thus contributing to lowering TG in plasma (Leaf, 2003; Seip et al., 1995). This process is accompanied by an increase in plasma HDL-C concentration, associated with the transfer of redundant surface lipids (phospholipids-PL and free cholesterol) from TG-rich lipoproteins to HDL particles (Seip et al., 1995).

The action of HL, hydrolyzing both TG and PL within HDL particles, alters the composition of these lipoproteins and accelerates their catabolism, so the exercise training-induced reduction of HL activity, evidenced by some authors, leads to a favorable elevation of plasma HDL-C concentrations (Leaf, 2003). In available literature, there is cumulative evidence indicating that endothelial lipase (EL), the enzyme synthesized mainly by vascular endothelial cells, plays a significant role in the modulation of the plasma HDL level (Jaye and Krawiec, 2004). Endothelial lipase, acting as the negative regulator of plasma HDL concentration, catalyzes the hydrolysis of HDL-PL and facilitates the clearance of HDL from circulation (Jaye and Krawiec, 2004). There are reports that variability in HDL responses to exercise training is dependent on EL gene polymorphism (Halverstadt et al., 2003), suggesting that this enzyme may be involved in the exercise-induced changes in plasma HDL concentration.

Many studies of the last decade revealed that angiopoietin like protein 3 (ANGPTL3), the liver-derived secretory factor, affects both plasma TG and HDL-C levels (Mattijssen and Kersten, 2012). Animal studies have evidenced that ANGPTL3 overexpression leads to a significant increase in plasma TG concentration, while the contrary effect, i.e. hypotriglyceridemia, has been shown in knockout mice (Angptl3−/−) that failed to express the Angptl3 gene (Fujimoto et al., 2006; Mattijssen and Kersten, 2012). The animal data indicate that this action of ANGPTL3 on plasma TG is determined by the ability of this protein to inhibit LPL and HL activities (Fujimoto et al., 2006; Mattijssen and Kersten, 2012). It was also found that in vitro, ANGPTL3 inhibited the phospholipase activity of endothelial lipase (EL), thus leading to an increase in both plasma HDL-C and HDL-PL concentrations (Mattijssen and Kersten, 2012; Shimamura et al., 2007). Moreover, ANGPTL3 was found to stimulate lipolysis of TG stored in adipose tissue, however, the underlying mechanisms of this effect and its significance for the accumulation of body fat are not fully understood (Fujimoto et al., 2006).

Although the role of ANGPTL3 as an inhibitor of LPL-mediated TG catabolism in humans has been confirmed by several genetic studies (Mattijssen and Kersten, 2012; Robciuc et al., 2013), the presumed positive relationship between plasma ANGPTL3 and TG was not found, indicating that the association of ANGPTL3 with the plasma TG-rich lipoprotein turnover is more complex (Hatsuda et al., 2007; Miida et al., 2008; Nakajima et al., 2010; Robciuc et al., 2010; Shoji et al., 2009). On the contrary, a positive correlation between plasma ANGPTL3 and HDL-C concentrations, commonly reported in human studies, supports the notion that the main metabolic function of ANGPTL3 in humans is the regulation of plasma HDL-C levels by inhibition of activities of both EL and HL (Miida et al., 2008; Nakajima et al., 2009; Shimamura et al., 2007; Shoji et al., 2009). It should be stressed, however, that most of the currently available knowledge regarding the role of ANGPTL3 in metabolic processes derives from experimental studies on individuals (Miida et al., 2008, Robciuc et al., 2013; Shoji et al., 2009), or animals (Fujimoto et al., 2006; Mattijssen and Kersten, 2012) with different abnormalities in lipid metabolism. The data on the role of AGPTL3 in healthy humans come mainly from studies of large population samples of a wide age range (Hatsuda et al., 2007; Moon et al., 2008, Robciuc et al., 2010; Stejskal et al., 2007), while only scarce data are available on young healthy individuals (Legry et al., 2009, Shimamura et al., 2007). Early negative alterations in lipid metabolism may appear in the youth, leading to the development of atherosclerosis in later life, although increased physical activity may, at least to some extent, counteract these unfavorable changes. Based on biochemical and epidemiological evidence of the role of ANGPTL3 in serum lipid turnover, we hypothesized that this protein may be involved in modification of the lipid profile induced by exercise-training. Given the lack of data regarding this issue, the main goal of the present study was to investigate the effects of regular participation in recreational physical activity on serum ANGPTL3 and selected lipid profile measures in young, apparently healthy female and male adults.

## Material and Methods

### Participants

Fifty healthy individuals (15 women and 35 men), recruited from students of the Academy of Physical Education (n=38) and the University of Silesia in Katowice (n=12), gave their written consent for participation in the study conducted in accordance with the Declaration of Helsinki, the experimental protocol of which was approved by the Ethics Committee at the Academy of Physical Education in Katowice, Poland (Certificate of approval No. 9/2011).

One group of participants consisted of twenty two students of the Faculty of Physical Education, who were not engaged in any competitive sport, but were highly active due to their participation in a compulsory physical education program with various components of physical fitness and voluntary training in a specific sport discipline. Their usual weekly physical conditioning program included different forms of physical exercise, such as football, swimming, jogging and resistance training. They practiced at least three times a week for a couple of hours each session (total duration range 6 to 10 hr per week), for at last three months preceding the study. This group was classified as recreational active subjects, including active females (AF, N=6) and males (AM, N=16).

The second group of participants consisted of age-matched, less active, relatively sedentary control groups of female (SF, N=9) and male (SM, N=19 ) individuals recruited from students of the Faculty of Theology (University of Silesia) and the Faculty of Tourism and Sports Management (Academy of Physical Education), not involved in any regular physical conditioning program, both classified as “sedentary” (S). All participants declared good health status, and most of them - as being non-smokers.

#### Experimental design

All participants from the physically active (A) and less active (S) groups reported to the physiological testing laboratory of the Academy in a fasted state in the morning, between 8.00 – 11.00 AM, for the anthropometric, metabolic and biochemical measurements. All laboratory procedures were carried out under similar environmental conditions (23–24°C, 50–60% RH).

### Procedures

Body height was measured to the nearest 0.5 cm in the standing upright position, without shoes, and body mass to the nearest 0.1 kg, to calculate the body mass index (BMI). Body composition (percent body fat - PBF, body fat mass - BFM, lean body mass - LBM, skeletal muscle mass - SMM, waist to hip ratio -WHR) was assessed using bio-electrical impedance analysis (In Body 220, Biospace).

After equilibration time of 10 min in a seated position, venous blood samples were drawn from the antecubital vein and processed using routine procedures to separate serum, then stored at −70°C until analyses. The concentration of serum ANGPTL3 was measured using a commercially available sandwich ELISA kit (Life Science Inc, cat.no. Uscn E91699Hu), highly specific for human ANGPTL3 (detection range 0.187–12ng/mL sensitivity 0.095ng/ml, Intra-Assay CV <10%, Inter-Assay CV <12%). The antibodies used in this ELISA kit were specific for human ANGPTL3 with no detectable cross-reactivities to human ANGPTL4. Serum concentrations of TG, total cholesterol (TC), HDL-C and glucose were assessed using commercially available diagnostic kits (TR210, CH201, CH203, GL2623, respectively) from Randox (UK). Concentrations of LDL-cholesterol (LDL-C) were calculated from TC, HDL-C and TG using the Friedewald formula. Additionally, to evaluate the risk for cardiovascular disease, traditional lipid ratios (TC/HDL-C, LDL-C/HDL-C, TG/HDL-C) with TC, TG, HDL-C and LDL-C in traditional units (mg/dL), as well as the Atherogenic Index of Plasma as the logarithmically transformed ratio of the molar concentration of TG to HDL-C (AIP=log_10_(TG/HDL-C) (Dobiašová et al., 2011), were calculated.

Furthermore, after completing anthropometric measurements and taking blood samples, the individuals from AM and FM groups performed an incremental ramp treadmill exercise, to estimate VO_2max_. The test was performed on a model LE 200 treadmill (Jaeger, Germany), beginning at 6.5 km/h and 0° inclination. Treadmill speed was increased by 1 km/h every minute (16 W per min) until reaching 12 or 14 km/h (respectively for women and men). Then, keeping the speed steady, the grade was set to 1.3°, and increased by 0.6° every min to reach increments in the workload (WR) of 8 W per min. The run was continued until volitional fatigue. During the test, a heart rate, minute ventilation (VE), oxygen uptake (VO_2_) and expired carbon dioxide (CO_2_) were continuously measured using a breath-by-breath system (MetaLyzer 3B-2R, Cortex, Leipzig, Germany).

### Statistical analysis

All variables were reported as mean ± SD. Given the relatively low number of participants, the comparison of anthropometric and biochemical indices between experimental groups was performed using the non-parametric Mann-Whitney test or the Kruskal-Wallis test for independent variables, respectively for women and men. In order to detect sex - related differences in measured variables in physically active (A) and inactive (S) groups of subjects, the non-parametric Mann-Whitney test was used. Relationships between variables were evaluated using the Spearman rank correlation test. The level of significance was set at *p* <0.05. All statistical analyses were performed using Statistica 9 software package (StatSoft, Tulsa, OK, USA).

## Results

The individual anthropometric variables of participants from both physically active (A) and less active (S) groups were mostly within the reference ranges for healthy subjects, except for PBF and BFM, which were higher in some men (N=7) from the sedentary group (SM). This prompted us to subdivide this group into two subgroups of normal-weight (SM-NW, N=12) with PBF ≤ 20%, and overweight individuals (SM-OW, N=7) with PBF > 20% (Table 1). It is worth noting that no significant differences were found between anthropometric variables recorded in less active, normal-weight (SM-NW) compared to the physically active (AM) men. On the contrary, significant differences between selected body composition measures were found in the overweight less active men (SM-OW) compared to less active normal-weight (SM-NW) or physically active (AM) men. As shown in Table 1, there were no significant differences in any anthropometric measures between less active (SF) and physically active (AF) groups of females.

In all experimental groups, most of the individual values of the lipid profile, including the common lipid ratios, AIP and glucose were within normal ranges (Grundy et al., 2004; Hanak et al., 2004; Rašlová et al., 2011). Among both female or male participants, there were no substantial between-group differences in serum ANGPTL3 levels and most other biochemical variables, except for serum TG that tended to be higher (p=0.09) in overweight males (SM-OW) compared to their physically active (AM) counterparts (Tables 2 and 3).

Significant sex-related differences in HDL-C (p<0.023) and TG (p<0.025) concentrations in favor of female subjects, accompanied by a tendency (p<0.051) toward higher serum ANGPTL3 in women, were found among the less active normal weight participants (SF and SM-NW). In physically active individuals (AF and AM), statistically significant sex-related differences were found in HDL-C (p<0.035) concentration (Tables 2 and 3). Moreover, significant sex-related differences were also found in the mean absolute (p<0.0005) and relative values of VO_2max_ (p<0.0005) recorded in physically active participants which, as presumed, were higher (4.37±0.38 l/min or 55.5±4.46 ml/kg/min) in men than in women (2.46±0.10 l/min or 42.5±3.21 ml/kg/min). Interestingly, a significant negative relationship (R= −0.559, p<0.03) between serum ANGPTL3 concentration and BFM was found in women, while in men, serum ANGPTL3 did not show any significant association with body composition measures.

## Discussion

The primary aim of the present investigation was to examine whether the participation in a recreational physical activity program would affect serum ANGPTL3 and the lipid profile in young healthy adults. Our pilot study, focused on the evaluation of differences in serum ANGPTL3 level between healthy, physically active subjects and their relatively sedentary counterparts, revealed that despite high inter-individual variability of this liver-derived secretory factor, there was a general tendency toward higher serum ANGPTL3 in women compared to men (p<0.07 for all normal-weight participants), but without significant differences related to their physical activity status.

High variation of individual serum ANGPTL3 levels in healthy individuals supports previous findings from population-based studies, however, there are discrepancies regarding the level of this protein in the blood. Some authors have reported that normal serum ANGPTL3 levels in healthy individuals range from 110 to 220 ng/ml (Miida et al., 2008; Moon et al., 2008; Shimamura et al., 2007), 200 to 450 ng/ml (Robciuc et al., 2010; Robciuc et al., 2013; Stejskal et al., 2007), to 570–875 ng/ml (Hatsuda et al., 2007; Shoji et al., 2009). These discrepancies may be due to differences in the type of biological material (lower values in serum than in plasma samples), or in the immunoassay analytical procedures (such as the type of antibodies or standards) used in the originally developed sandwich ELISA system (Miida et al., 2008; Robciuc et al., 2010; Shimamura et al., 2007) or in the commercially available assay kits (Hatsuda et al., 2009; Shoji et al., 2009; Stejskal et al., 2007) each yielding somewhat different results. Moreover, based on the data from animal studies (Mattijssen and Kersten, 2012), it seems reasonable to assume that both nutritional and hormonal factors may affect the ANGPTL3 level, but in our study and in most other human studies, the contribution of these factors to the circulating ANGPTL3 level was not estimated. Most similar to our results are the data presented by Robciouc et al. (2010), who found that in a healthy Finnish population the average serum ANGPTL3 was 347.88 ±159.4 ng/ml in men and 388.6 ±20.6 ng/ml in women, and this difference was close to significance (p<0.08). Similar statistically significant sex-related differences in ANGPTL3 levels in a large population sample of individuals aged 46 to 61 years were reported by Mehta et al. (2014).

In contrast, other authors have not found any sex-related differences in the serum ANGPTL3 level in the Czech (Stejskal et al., 2007) and Japanese population (Hatsuda et al., 2007), however, those studies were conducted with middle-aged or older adults, in whom sex-dependent differences may have been less pronounced than in younger individuals.

Numerous animal studies have revealed that ANGPTL3 protein affects both TG and HDL-C concentrations (Mattijssen and Kersten, 2012), and the data from human studies suggest that ANGPTL3 is associated more strongly with activities of EL and HL, the enzymes involved in regulation of the HDL-C metabolism (Miida et al., 2008; Moon et al., 2008; Nakajima et al., 2010; Robciuc et al., 2010; Shimamura et al., 2007). The contribution of ANGPTL3 to maintaining serum HDL-C levels was confirmed by Miida et al. (2008), who found that the pharmacologically induced decrease in ANGPTL3 resulted in a subsequent drop in the plasma HDL-C level. Similarly, Shoji et al.(2009) showed that low levels of HDL-C (means 38.9 mg/dl) in patients with uremic dyslipidemia were accompanied with decreased serum ANGPTL3, that reached only about half (40–50%) of the levels recorded in the age-matched control group, having significantly higher HDL-C levels (means 53.6 mg/dl). Consistent with this finding are the reports of other authors (Mattijssen and Kersten, 2012; Robciuc et al., 2013), who found that the genetic mutation of the Angptl3 gene, leading to the complete absence of circulating ANGPTL3, was associated with a dramatically low level of HDL-C. In contrast, the abnormally high frequency of increased ANGPTL3 levels (>362 ng/ml), associated with extremely high levels of HDL-C (above 90 mg/dl), was found to prevail in Japanese hyper-alpha-lipoproteinemic subjects (Moon et al., 2008). Moreover, a high correlation of ANGPTL3 with HDL-C, but not with cholesteryl ester transfer protein (CETP) in those subjects, suggests that ANGPTL3, the inhibitor of EL, is a stronger modulator of HDL-C than CETP.

Contrary to reports showing a marked association between HDL-C and ANGPTL3 levels, in the present study we did not find a significant relationship between these variables. The reason of this discrepancy may be that our data were collected from a small group of young, normolipidemic subjects with relatively small inter-individual variability in the HDL-C level. It is worth adding that we have recently recorded (our unpublished data) markedly higher levels of ANGPTL3 (432.6±10.5 ng/ml), associated with a more favorable lipid profile (TG - 0.7±0.1 mmol/l; HDL-C - 1.4±0.2 mmol/l) in highly endurance-trained men (N=8, mean age 23.4±4.2 yr, BMI 21.1±1.6 kg/m2, PBF 11.6±2.4%, VO_2max_ 62±2.8 ml/kg/min). This seems to support the hypothesis of the involvement of ANGPTL3 in the exercise-induced improvement of the lipid profile, more evident following endurance than resistance training (Durstine et al., 2001; Tambalis et al., 2009).

An interesting finding of our study was that the expected sex-related differences in the concentration of HDL-C (p<0.005) were accompanied by a trend toward higher levels of ANGPTL3 in women compared to men. It is well known that higher levels of HDL-C in women are consequent to sex-related differences in the activities of both LPL and HL. Women, characterized by higher selective deposition of femoral fat compared to men, have higher femoral adipose tissue LPL activity and higher HDL-C due to an efficient pathway clearing TG-rich lipoproteins from circulation (Després et al., 2008). On the other hand, the activity of HL, that is approximately twice lower in women than in men, has been shown to be regulated by endogenous sex steroids (estrogens suppress, while androgens increase HL activity) (Deep et al., 2003). Given the role of ANGPTL3 in suppression of HL activity (Nakajima et al., 2010), it can be hypothesized that ANGPTL3 may also contribute to sex-related variability in HDL-C, but, to our knowledge, there are no available data regarding the impact of estrogens on ANGPTL3 expression.

An active lifestyle and high physical fitness are recognized as key factors affecting overall risk of cardiovascular disease, partly due to favorable changes in the blood lipid and lipoprotein profile. Based on both routine lipid profile measures (TG, HDL-C, LDL-C, T-C) and traditional lipid ratios such as T-C/HDL-C, HDL-C/LDL-C and TG/HDL-C, all normal weight participants of the present study had a normal lipid profile and glycemia, as well as favorable anthropometric indices not suggesting risk of CVD.

Equally important is our finding that in most participants, the atherogenic index of plasma (AIP) adopted the negative values, with a tendency toward lower levels in physically active compared to their less active counterparts. Given that AIP is closely and inversely associated with particle size of LDL-C and HDL-C, as evidenced by Dobiášová et al. (2011), it may represent a reliable biomarker of CVD risk. The negative AIP values recorded in most participants reflect prevalence of large, buoyant and less atherogenic LDL and HDL particles in plasma and a low risk of CVD. However, it is worth noting that only in two male volunteers from the relatively sedentary and overweight (SM-OW) group, the AIP values were above the threshold of high risk category (AIP>0.21) (Rašlová et al., 2011), while in all remaining cases, the AIP did not reach the low risk limit (AIP<0.11). Moreover, only in the same two cases the TG/HDL-C ratio exceeded the level of 3.8, suitable for predicting LDL phenotype B, characterized by predominance of small dense LDL particles (Hanak et al., 2004). Lower levels of the TG/HDL-C ratio and AIP in physically active, compared to less active participants, strongly support the view of beneficial health-promoting effects of regular participation in recreational activities.

Interestingly, no associations were found between ANGPTL3 levels and the lipid profile, lipid ratios and anthropometric measures, which is consistent with observations of other authors (Hatsuda et al., 2007; Miida et al., 2008; Robciuc et al., 2010; Shimamura et al., 2007). However, contrary to other researches (Popovic et al., 2010; Scheers et al., 2007), in the present study we found only modest differences in the lipid profile and lipid ratios, related to physical activity status of our subjects. These changes were accompanied with slight differences in serum ANGPTL3 levels between relatively sedentary individuals and their more active peers. The observed lack of the expected alterations in the lipid profile can be attributed both to the mode and volume of exercise training applied by our physically active students. Their habitual training program contained both an endurance and resistance type of muscular activity, while a health oriented and lipid lowering training regime is based mainly on typical endurance activities (Durstine et al., 2001). Additionally, the intensity of training workouts was probably too low to induce the pronounced changes in the lipid profile (Durstine et al., 2001; Tambalis et al., 2009). To the best of our knowledge, there are no data on physical activity-related changes in the ANGPTL3 level, which implies that further investigations in this area are needed.

The main limitations of this study were its cross-sectional design, relatively small sample sizes, and lack of information on the subjects’ dietary intake as well as the actual physical workloads, which limit the generalisability of research findings.

In conclusion, regular participation of young adults in recreational physical activities is not associated with marked alterations in the level of circulating, liver derived ANGPTL3 and lipid profile measures, although independent of physical activity status, serum contents of ANGPTL3 and HDL-C are higher in females than in males. Lower levels of the TG/HDL-C ratio and AIP in physically active compared to relatively sedentary participants, reflecting predominance of large, buoyant LDL particles, strongly support the view of beneficial health-promoting effects of regular participation in recreational activities.

## Figures and Tables

**Table 1:** Anthropometric characteristics of participants (mean±SD)

Variables	Males			Females	
	Sedentary	Physically active		Sedentary	Physically active
	SM-NW (n=12)	SM-OW (n=7)	AM (n=16)	SF (n = 9)	AF (n = 6)
Age (years)	21.8±2.3	25.1±2.0	23.4±2.0	20.6±0.7	24.3±1.4*
Body height (cm)	183.5±5.8	177.1±6.0	182.1±6.7	165.4±4.2	164.2±4.6+
Body mass (kg)	74.6±7.7	78.8±13.3	79.2±8.0	56.2±6.8+	57.9±3.5+
BMI (kg/m^2^)	22.1±2.0	25.0±3.3	23.9±1.8	20.5±2.0	21.5±1.2+
PBF (%)	14.9±3.8	25.5±3.0*	12.4±3.1#	24.6±4.1+	23.6±1.8+
BFM (kg)	11.3±3.46	20.1±4.3*	9.9±3.0#	14.0±3.7	13.7±1.5+
LBM (kg)	63.3±5.5	58.7±10.1	69.3±6.7#	42.2±3.9+	44.2±2.6+
SMM (kg)	35.7±3.3	32.9±6.1	39.6±3.9#	23.0±2.4+	24.2±1.6+
SMM/body mass (%)	47.9±2.1	41.7±1.9*	50.0±1.9#	41.1±2.3+	41.9±1.0+
LBM/BFM ratio	6.2±2.3	3.0±0.5*	7.5±2.2#	3.2±0.7+	3.3±0.3+
WHR	0.83±0.02	0.88±0.03*	0.85±0.03	0.79±0.03+	0.79±0.02+

SM-NW-sedentary normal-weight males, PBF<20%; SM-OW-sedentary overweight males, PBF>20%; AM-physically active males, PBF<20%; SF-sedentary females, AF - physically active females, BMI - body mass index, PBF - percent body fat, BFM - body fat mass, LBM - lean body mass, SMM - skeletal muscle mass, WHR - waist to hip ratio

* significantly (p<0.05) different from respective values in the SM-NW or AM groups

# significantly (p<0.05) different from the SM-OW group

+ significantly (p<0.05) different in SF or AF groups compared to SM-NW or AM groups, respectively.

**Table 2:** Lipid profile, serum ANGPTL3 and glucose concentrations in male participants (mean ±SD)

Variables	Males			Normal Range or URL
	Sedentary		Physically active	
	SM-NW (n =12) PBF <20%	SM-OW (n =7) PBF >20%	AM (n =16) PBF <20%	
T-C (mmol/l)	3.7±0.8	3.2±1.1	4.3±1.0	3.0–5.0
HDL-C (mmol/l)	1.3±0.2	1.3±0.3	1.4±0.2	>1.0
LDL-C (mmol/l)	1.9±0.8	1.5±1.1	2.4±0.6	<2.6
T-C/HDL-C ratio	3.0±0.8	2.5±0.6	3.0±0.6	<4.0
LDL-C/HDL-C ratio	1.5±0.7	1.2±0.7	1.7±0.6	<4.5
TG (mmol/l)	0.95±0.09	1.27±0.12	0.91±0.08	<1.7
TG/HDL-C ratio	1.8±0.7	2.4±1.3	1.5±0.4	<3.8^a^
AIP	−0.14±0.17	−0.003±0.24	−0.21±0.12	<0.11^b^
ANGPTL3 (ng/ml)	271.5±94.9	252.6±88.9	299.8±96.4	n/a
Glucose (mmol/l)	4.9±0.8	4.9±0.5	4.9±0.4	3.4–5.5

SM-NW - sedentary normal - weight males, SM-OW - sedentary overweight males, AM - physically active males

a according to Hanak et al. (2004);

b according to Rašlova et al. (2011);

n/a – not available

**Table 3:** Lipid profile, serum ANGPTL3 and glucose concentrations in female participants (mean ±SD)

Variables	Females		Normal range or URL
	Sedentary (n=9) SF	Physically active (n= 6) AF	
T-C (mmol/l)	4.1±0.8	4.2±1.1	3.0–5.0
HDL-C (mmol/l)	1.6±0.3	1.8±0.4	>1.2
LDL-C (mmol/l)	2.0±0.7	2.2±0.9	<2.6
T-C/HDL-C ratio	2.7±0.6	2.9±1.3	<4.0
LDL-C/HDL-C ratio	1.3±0.5	1.3±0.5	<4.5
TG (mmol/l)	1.2±0.3	1.0±0.4	<1.7
TG/HDL-C ratio	1.8±0.7	1.2±0.6	<3.8^a^
AIP	−0.13±0.16	−0.29±0.17	<0.11^b^
ANGPTL3 (ng/ml)	355.6±96.7	338.4±143.2	n/a
Glucose (mmol/l)	4.8±0.3	4.7±0.5	3.4–5.5

SF - sedentary females, AF - physically active females

a according to Hanak et al. (2004);

b according to Rašlova et al. (2011);

n/a – not available
